# Applications of Xylan Derivatives to Improve the Functional Properties of Cellulose Foams for Noise Insulation

**DOI:** 10.3390/polym15244648

**Published:** 2023-12-08

**Authors:** Silviu Marian Nastac, Petronela Nechita, Maria Violeta Guiman, Mirela Roman, Ioan Calin Rosca

**Affiliations:** 1 Research Center for Mechanics of Machines and Technological Equipment, Engineering and Agronomy Faculty in Braila, “Dunarea de Jos” University of Galati, 810017 Braila, Romania; mirela.roman@ugal.ro; 2 Faculty of Mechanical Engineering, “Transilvania” University of Brasov, 500024 Brasov, Romania; icrosca@unitbv.ro

**Keywords:** cellulose fiber, foam-forming, xylan derivatives, noise insulation, wettability, sound absorption, sound transmission loss

## Abstract

Cellulose-based foams present a high potential for noise insulation applications. These materials are bio-degradable, eco-friendly by both embedded components and manufacturing process, have low density and high porosity, and are able to provide good noise insulation characteristics compared with available petroleum-based foams currently used on a large scale. This paper presents the results of some investigations performed by the authors in order to improve the functional characteristics in terms of free surface wettability and structural integrity. Native xylan and xylan-based derivatives (in terms of acetylated and hydrophobized xylan) were taken into account for surface treatment of cellulose foams, suggesting that hemicelluloses represent by-products of pulp and paper industry, and xylan polysaccharides are the most abundant hemicelluloses type. The investigations were mainly conducted in order to evaluate the level to which surface treatments have affected the noise insulation properties of basic cellulose foams. The results indicate that surface treatments with xylan derivatives have slowly affected the soundproofing characteristics of foams, but these clearly have to be taken into account because of their high decrease in wettability level and improving structural integrity.

## 1. Introduction

Foam-formed cellulose biomaterials represent a promising technology for developing lightweight and sustainable materials, which are in high demand for packaging, cushioning, and insulation applications [1,2,3,4]. Cellulosic foams, derived from cellulose fibers, exhibit intriguing properties, including extremely low density [5,6,7], high fluid permeability [8,9,10], and effective sound and heat insulation [11,12,13,14,15]. In line with this trend, Cucharero et al. investigated sound-absorbing materials produced using foam-forming methods based on hardwood and softwood pulps as raw materials [16]. The foams exhibited sound absorption characteristics comparable to those of typical porous materials, commonly employed in contemporary soundproofing applications on a large scale. The findings from this study offer valuable insights into the optimization of wood-based noise insulation materials. Jahangiri et al. also proposed a novel biodegradable, low-density porous material based on wood fibers and produced in foam-laid media [17,18,19]. An experimental setup based on a three-microphone impedance tube was employed to investigate acoustical properties concerning the potential effects of various parameters (e.g., foam air content, thickness, porosity, and consistency of the foam–fiber solution).

In recent years, the authors of this article have developed cellulose fiber-based porous lightweight materials using foam-forming techniques and various types of cellulose fibers as raw components [20]. These materials have primarily been applied for noise insulation [21,22,23,24], thermal insulation [25], and cushioning/packaging purposes [26,27]. Experimental investigations have generally shown that cellulose foams are able to provide insulation characteristics similar to or better than petroleum-based materials/composites, commonly used as a protective solution in most cases.

Despite the fact that wet-laying is a mature technology widely applied in specific industries, it typically involves the use of large volumes of water and is energy intensive [28]. In this regard, the foam-laying method offers a substantial reduction in water consumption during the fibrous network formation process and helps decrease the water content of the non-wovens produced before drying, thereby achieving a reduced energy demand [29,30,31,32,33]. An interesting and useful overview of the foam-forming technique was presented by Hjelt et al. in their paper [34], where they addressed both fundamental foam properties and practical forming methods. They also demonstrated how the material characteristics can be affected by foam–fiber interactions. Furthermore, the potential material characteristics were compared against key requirements in typical product applications. Another useful survey regarding applications of foam-formed cellulose-based materials for cushioning packaging was provided in [35], where the authors presented a comprehensive state-of-the-art overview of the materials involved, available methods, and the main properties of these foams.

Scientists have also focused on non-wood fibers as a viable alternative for the pulp and paper industry. In their article [36], Abd El-Sayed et al. showed that actual high-tech innovation has made non-wood more reasonable than wood as a raw material for the papermaking industry, even in countries with acceptable wood sources, due to environmental concerns. The foam-forming method also presents high interest in the exploitation of proven design concepts for advanced material solutions [37,38]. The authors of [39] proposed a foam structure that presents high-yield stress in the primary direction and excellent thermal insulation. In addition, cellulose-based foams offer an unlimited creative space for the design of green functional materials with a wide range of energy-related applications. Thus, the work [40] demonstrates that cellulose-based foams can exhibit solid–liquid phase change functionality and shows the versatility of the foam-forming process of cellulose-based materials in order to accommodate physical functionalities in materials with complex architectures. The authors developed foams that are recyclable, industrially scalable, and can be exploited as heat storage materials.

Regarding the subtle fiber–bubble interactions, the research [41] demonstrates that these interactions provide a suitable tool that can be used to alter both structural and mechanical material properties of foam-formed cellulose-based materials. A large ensemble of studies was developed within the area of soundproofing, being mainly focused on materials or composites suitable for applications in noise insulation practice [42,43,44,45,46].

Different types of fiber raw sources are used worldwide, related to available wood resources for a certain geographical area. For example, Malekzadeh et al. [47] demonstrated the potential of bamboo fiber-based porous materials as low-cost, lightweight structural materials. The authors of paper [5] present a new method to produce cellulose fiber-based lightweight materials, using eucalyptus pulp as raw material and fibers partially hydrolyzed with sulfuric acid. The advantage of this method is justified by a drying step easily performed at mild temperatures in a convection oven, thus eliminating the need for more sophisticated drying techniques. Moreover, the proposed method does not require surfactants or special foam-forming equipment.

Using pineapple leaf fibers with paper waste, a research group developed composites with good performances related to sound absorption and impact strength [48]. Ultra-lightweight cellulose foams with good mechanical properties were proposed in article [49]. The authors have observed that micro-pores (bubbles) inside the wet foams weakened the mechanical properties but increased these properties in the dried foams. Their investigations revealed good compressive strength of dried foams, which exhibited great potential for further development and comprehensive utilization of cellulose. Other groups of raw sources for composites with very interesting characteristics and potential applications in sound insulation practice are cork sheets [50,51,52,53] and corn stalk fibers [54].

The required strength of cellulose-based foams can be achieved by optimally combining fibers and fines of different length scales [55]. In addition, the elasticity can be improved by adding polymers, which accumulate at fiber joints and help the network structure recover after compression. Within their study [55], the authors showed that structure and elastic properties were sensitive not only to the raw materials but also to the elastomer stiffness and foam properties. Additionally, an improved strain recovery makes the developed cellulose-based materials suitable for various applications, such as padding for insulation purposes.

By incorporating different additives (e.g., chitosan, cationic polyacrylamide), Meiyan et al. improved some functional properties of pulp foam (in terms of physical strength, fire-retardance, thermal insulation, antibiosis, and sound absorption) [56]. Interesting biodegradable chitosan-based foams were also proposed in study [57]. New bio-based packaging materials present high interest in replacing conventional fossil-based products for a more sustainable society [58]. The cellulose fiber-based foams containing chitosan provide both good water stability and good antibacterial and antifungal properties, demonstrating the feasibility of bio-based foam material with desired characteristics. Hereby, these foams can enable an interesting low-density packaging composite with protective mechanical and microbial properties without using any toxic compounds [56,57,58]. In addition, Seppänen et al. evaluated the wet strength properties of foam-formed fiber-based materials [59]. Novel lightweight cellulose fiber-based composites, embedding various strength-enhancing polymeric and fibrillar components, were developed by Pöhler and collaborators using the foam-forming technology [60]. During their research, increasing both inter-fiber bond strength and local material density was attempted.

As they are the second class after cellulose, hemicelluloses are heterogeneous polysaccharides that exist in almost all cell walls of lignocellulosic biomass. Xylan polysaccharides are the most abundant hemicellulose type, representing about 20–35%, mainly in hardwood and annual plants (wheat straw, corn stalks, and cobs) and also as a by-product of the pulp and paper industry [61,62].

Although it exists in large quantities, the industrial application of xylan hemicellulose is still limited. It is used to obtain the xylitol and bio-fuels by biological conversion of sugar, starch, and vegetable oils. In the packaging industry, xylan hemicellulose is used to improve the strength and biodegradability properties of plastic materials [63]. Generally, the limitation of industrial applications for xylan hemicellulose is due to its high hydrophilicity as a result of a larger number of OH groups in the structural unit. To enhance its hydrophobicity and processability by reducing the H-bonds, the hydrophobic groups are attached to the hemicellulose chains by different methods of chemical modification. Therefore, chemical modification with hydrophobic moieties was applied in order to improve the function of hemicelluloses for packaging and coating applications [64]. In addition, cationic xylan derivatives have a wide range of applications in the papermaking industry, such as cellulose fiber strength additives, flocculation aids, and antimicrobial agents [63].

Considering the good compatibility of xylan with cellulose fibers, this paper aims to utilize xylan derivatives to enhance the free surface wetting and structural integrity of cellulose fiber-based foams. This improvement enables the materials to be incorporated into sound insulation applications. To achieve this goal, native hardwood xylan hemicellulose, along with its acetylated and hydrophobized derivatives, was applied as coatings in a thin layer to treat the free surface of cellulose foams. The coated samples underwent analysis to assess noise insulation characteristics and performance, aiming to evaluate the impact of xylan-based coatings on soundproofing capabilities. This evaluation involved a comparison with untreated cellulose-based samples and a few samples of commercially available petroleum-based materials commonly used in sound insulation.

The originality of this study arises from several aspects: (i) the novel use of cellulose fiber foams in soundproofing applications; (ii) the imperative need to ensure appropriate wetting and surface structure integrity properties for these materials, enabling their practical utilization as noise insulation panels; and (iii) the utilization of hemicelluloses, by-products of the pulp and paper industry, with xylan polysaccharides being the most abundant hemicellulose type.

Essentially, based on available scientific reports, this type of surface coating (utilizing xylan derivatives) has not been thoroughly analyzed. The effects of this treatment must be rigorously evaluated concerning changes in noise insulation characteristics to validate its functional capability in practical applications. In addition, it has to be underlined that some other research groups, which have analyzed the necessity of foam-free surface coating, generally used synthetic petroleum-based solutions or with very complicated production technology (also environmental pollutants). Therefore, given the previously mentioned advantages, xylan-based derivatives offer a viable solution for enhancing the hydrophobicity and surface structure integrity of cellulosic foams.

## 2. Materials and Methods

### 2.1. Materials

The proposed porous lightweight materials were developed in a foam medium using virgin softwood cellulose fibers. Bleached softwood cellulose (typically providing 400–800 µm average fiber length and 15–35 µm diameter) was used as a raw fiber source [21,22,27]. Cellulose fibers, with 60 and 43 °SR beating degrees (according to the Schopper–Riegler evaluation method), and slurry pulp with 1.98% consistency were adopted within this research. The anionic surfactant used for foam forming was sodium dodecyl sulfate (SDS), commonly utilized in commercial regular cosmetics production. Based on these components (slurry pulp and surfactant), two types of foam-formed cellulose-based lightweight foams were obtained (please see details within Section 2.2 and Section 3.1) [21,22,23,24,27,35].

Xylan hemicellulose with a molecular mass of (132)n from beechwood was purchased from Carl Roth Company, Karlsruhe, Germany, and used as received.

Acetylated xylan was obtained in laboratory conditions according to the procedure described by the authors in previous papers [26,65]. The procedure involved the esterification process of native xylan with acetic anhydride in two stages, at 50 °C for 1 h and a molar ratio of acetic anhydride to functional hydroxyl groups in the structural unit of xylan about 8:1. The degree of substitution was about 0.48 [26,65].

Hydrophobized xylan was obtained by reaction of native xylan with long chain anhydrides as alkyl ketene dimers at 20 °C and 24 h of magnetic stirrer at 1500 rpm. Alkyl Ketene Dimer of milky white liquid, odorless, total solids of 16.2% and viscosity of 4 cPs/t = 25 °C (as a commercial product, AquapelTM 210D—Solenis, Wilmington, DE, USA), was used for xylan hydrophobization [26,65]. All the other chemical reagents for esterification reaction (acetic anhydride, acetic acid, and sulfuric acid) have had analytical purity.

Both native xylan and xylan derivatives were used as water dispersion of 2.5% for coating by pulverization in a thin layer (about 5 g/m^2^) on the surface of different samples of cellulose foams.

**Figure 1:**
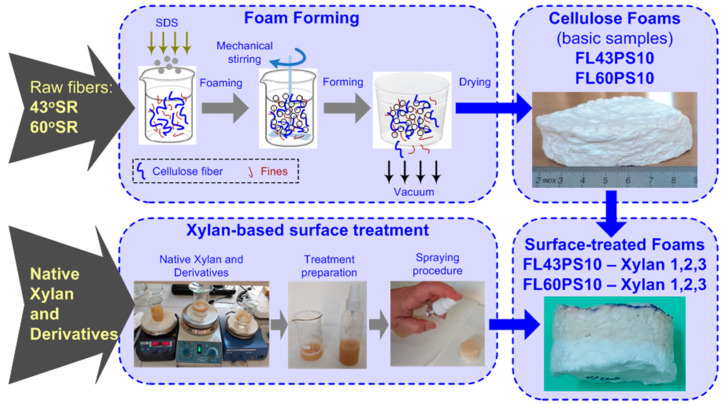
Schematic diagram of sample preparation, based on cellulose fiber foam-forming technique and xylan and xylan derivatives for free surface treatments.

A schematic diagram of sample preparation is provided in Figure 1. The upper section of this scheme presents the basic procedure to obtain cellulose fiber-based foams [21,22,23,24,27,35]. At the same time, the lower section presents relevant aspects regarding the xylan-based coatings of foam-free surfaces in terms of magnetic stirrers used for homogenization of native xylan and xylan derivatives water dispersion, preparation of dispersion for sample surface treatment, and an example of xylan treatment laid on sample surface using the spraying method.

Four different commercially available petroleum-based materials currently used in sound insulation were also considered in order to provide a suitable reference for analyzing the performances of cellulose-based materials (with and without xylan-free surface treatments). These materials include expanded polystyrene (EPS), extruded polystyrene (XEPS), polyurethane (PU), and expanded polyethylene (EPE).

### 2.2. Methods within Sample Forming Procedure

The cellulose fibers (resinous virgin cellulose fibers with 1.98% consistency) were soaked overnight (approx. 24 h) using distillate water with 1% sodium hydroxide (1 N concentration). Next, the slurry pulp was mixed using high shear velocity up to 2200 rpm for 20 min in order to facilitate air entraining. During the agitation process, a controlled percentage of surfactant (relative to the pulp weight) was added to obtain a suitable foam medium.

The authors have aimed to obtain and analyze two types of foam materials based on two different beating degrees of raw pulp with constant air content. Thereby, 4% of surfactant was adopted relative to the fiber weight (this is the mid value of the surfactant percentage range used in previous investigations [21]).

The suspension of fibers and foam was filtered and dewatered using a Buchner funnel and a sample holder with an inner diameter correlated to that required by equipment used in acoustical investigations (e.g., 100, 72, and 28.5 mm). A filter paper was used at a sample bottom, in addition to the filtering system of the Buchner funnel, aiming to obtain a sample surface as flat as possible. The filtering was developed at a low vacuum level for approx. 20 min, following both a suitable dewatering and the sample structural integrity. After dewatering, the samples were carefully transferred to the drying table, aiming to avoid structural integrity loss. Samples were dried at room temperature (around 22 °C) and 50–60% relative humidity for 24 to 48 h (please see details within Section 3.1) [21,22,23,24,27,35].

### 2.3. Methods within Contact Angle Evaluation

In order to evaluate the hydrophobicity of cellulose foams in terms of free surface wettability, the static water contact angle was measured according to the T-458 cm-04 Standard by static sessile drop method [66]. This procedure used the Ossila^®^ contact angle goniometer (Ossila BV, Leiden, The Netherlands), which is equipped with a high-resolution digital camera and suitable software for recording and processing the results.

The samples were placed on the test table, and water drops (approx. 5 μL) were deposited onto its surface with a micro-syringe. The value of the contact angle was recorded after 5 s of water–substrate contact time on each sample. The measurements were repeated at five different locations on the cellulose foam surface, and the average contact angle values were reported (please see details within Section 3.2).

### 2.4. Methods within FT-IR Investigations

The structural characteristics, which highlight the presence of specific chemical groups in the modified xylan samples, were analyzed using a Nicolet iS50 FT-IR spectrometer (Thermo Scientific, Waltham, MA, USA) equipped with an attenuated total reflection (ATR) accessory and a diamond crystal plate, in transmission mode. The spectrometer was placed in a temperature-controlled room (21 ± 2 °C). Infrared spectra were measured within the spectral range 4000–400 cm^−1^ at 2 cm^−1^ spectral resolution and 32 background/sample scans using OMNIC^TM^ software (Thermo Fisher Scientific Inc., Waltham, MA, USA). The background spectrum was collected by taking air as a reference before each measurement, and the diamond crystal plate was cleaned with alcohol.

### 2.5. Methods within Noise Insulation Investigations

**Figure 2:**
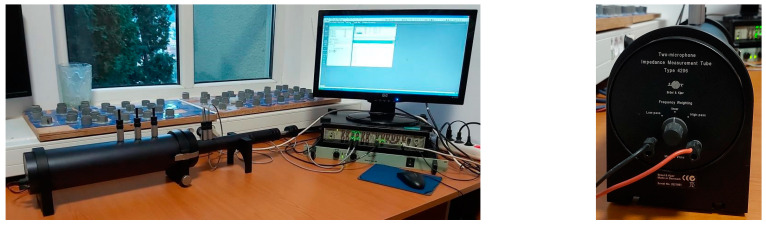
The Impedance Tube Kit Type 4206^®^ (Brüel&Kjær) used for acoustical investigations.

The foam samples, both untreated and xylan-based surface treated, were analyzed in order to estimate the acoustic insulation capabilities in terms of absorption and reflection coefficients and acoustic impedance, with all these parameters being evaluated at a normal incidence angle. Experimental investigations were conducted using an Impedance Tube Kit Type 4206^®^ (Brüel&Kjær Sound and Vibration Measurement A/S, Nærum, Denmark), with a 29 mm diameter sample holder and a two-microphones setup configuration (Figure 2). The frequency range enabled by this tube is 500–6400 Hz (according to the technical specifications). A sound source (that generates broadband, stationary random sound waves) is mounted at one end of the impedance tube, and the sample of material is placed at the other end. The propagation, contact, and reflection result in a standing-wave interference pattern due to the superposition of forward- and backward-traveling waves inside the tube. Measuring the sound pressure at two fixed locations and evaluating the complex transfer function using a two-channel frequency analyzer result the sound absorption, complex reflection coefficients, and the normal acoustic impedance of the tested material within the sample holder. The measurements were developed based on the transfer function method and testing method described in ISO 10534-2 [67] and ASTM E1050-12 International Standards [68].

Acoustic measurements were made with two 1/4″ Pressure-field Condenser Microphones Type 4187^®^ (Brüel&Kjær Sound and Vibration Measurement A/S, Nærum, Denmark), which are supplied with a tube kit and are specially designed to reduce errors due to pressure leakage at high frequencies.

Data acquisition and primary processing were managed with the software platform PULSE Acoustic Material Testing Type 7758^®^ (Brüel&Kjær Sound and Vibration Measurement A/S, Nærum, Denmark). Advanced computational developments were performed using a set of Matlab^®^R2018b (MathWorks, Natick, MA, USA) based applications.

## 3. Results

### 3.1. Cellulose-Based Foam Formed Samples with Xylan-Based Treatments

**Figure 3:**
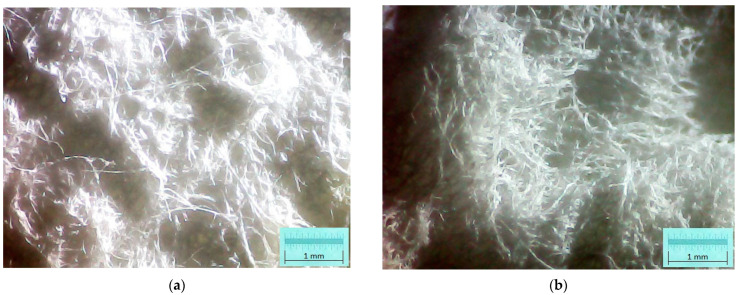
Optical micro-photographs of cellulose-based foam-formed samples surface: (**a**) foam based on long fibers with 43 °SR beating degree; (**b**) foam based on long fibers with 60 °SR beating degree.

According to the methods presented in Section 2.2 and using the raw materials presented in Section 2.1, two types of resinous fibers cellulose-based foams were made: one type with a 43 °SR beating degree and another with a 60 °SR beating degree. Microphotographs within Figure 3 depict the micro-fibrous structure of external surfaces for each sample type. These images were acquired using optical microscopy technique, with transmitted dead white light, based on DELTA Optical Three-Ocular Microscope model SZ-450T^®^ (Delta Optical, Minsk Mazowiecki, Poland), and Bresser MikrOkular Full HD Digital Camera^®^ (Bresser GmbH, Rhede, Germany). Each image provides a dimensional grid, suitably marked on the picture and gained by a standard calibration grid lamella.

**Figure 4:**
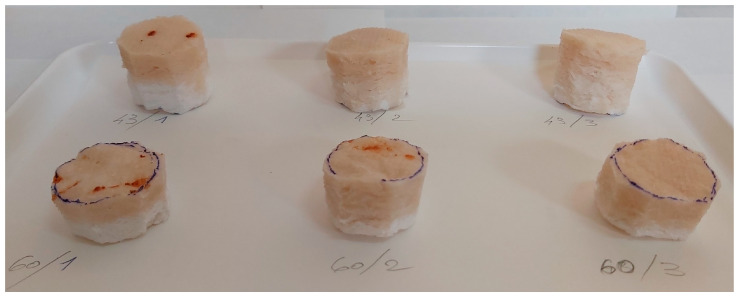
Photo of wet-state samples just after the xylan-based treatments (see text for sample coding details).

The samples with applied xylan-based surface treatments were depicted in Figure 4, where the sample codes have the following meanings: (43, 60)/(1, 2, 3)—base foam with 43 or 60 °SR beating degree and treatment with native xylan (1), AKD hydrophobized xylan (2), and acetylated xylan (3). The pictures in Figure 4 were acquired just after the xylan spraying procedure.

**Figure 5:**
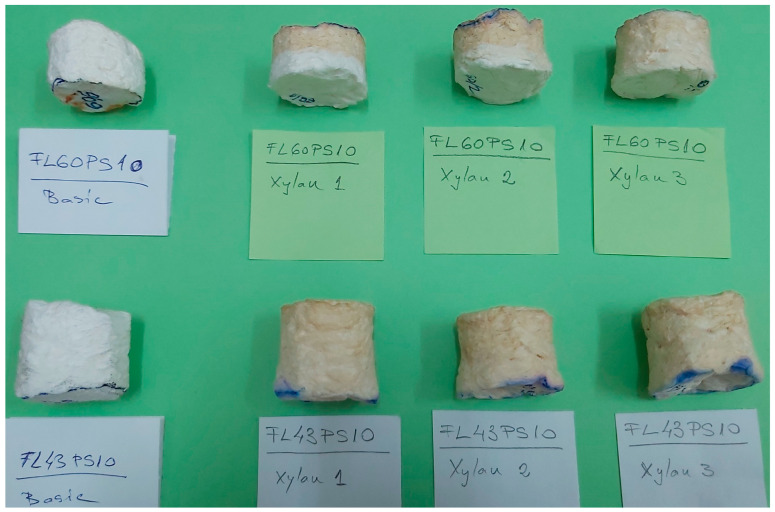
Photo of dry-state samples 48 h after the xylan-based treatments (see text for sample coding details).

Surface-treated samples were dried on filter paper (in order to take over the possible xylan leakages through samples) at room temperature (approx. 22 °C) for 48 h. Dry-state samples (ready-to-use foams) were presented in Figure 5, where sample codes have the following meanings: FL(43, 60) PS10—Long Fiber with 43 or 60 °SR beating degree, and 4% surfactant (SDS); Xylan (1, 2, 3)—native xylan (1), AKD hydrophobized xylan (2), and acetylated xylan (3). The image within Figure 5 also contains the reference foams (denoted as “Basic”), enabling a comparative analysis between samples with and without surface treatments.

In order to enable the analyses of xylan–foam interactions and the effects of treatment on the foam structure, a longitudinal section on samples was considered. Thus, a thin slice of 4 mm thickness was cut from each sample. These sectional samples were analyzed with both reflected and transmitted dead white light (without optical magnification) and with optical transmitted light microscopy.

**Figure 6:**
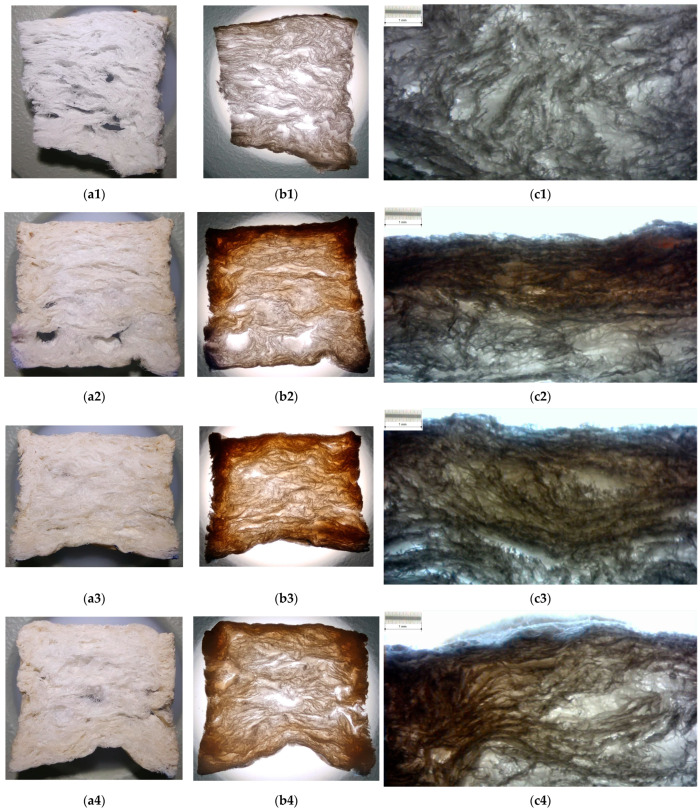
Images of longitudinal sections into samples based on 43 °SR beating degree: (**a**) photo on reflected light; (**b**) photo on transmitted light; (**c**) optical microphotograph of xylan–material interface (transmitted light, etalon scale marked on picture); (**1**) basic samples without xylan surface treatment; (**2**) samples with native xylan surface treatment; (**3**) samples with AKD hydrophobized xylan surface treatment; (**4**) samples with acetylated xylan surface treatment.

**Figure 7:**
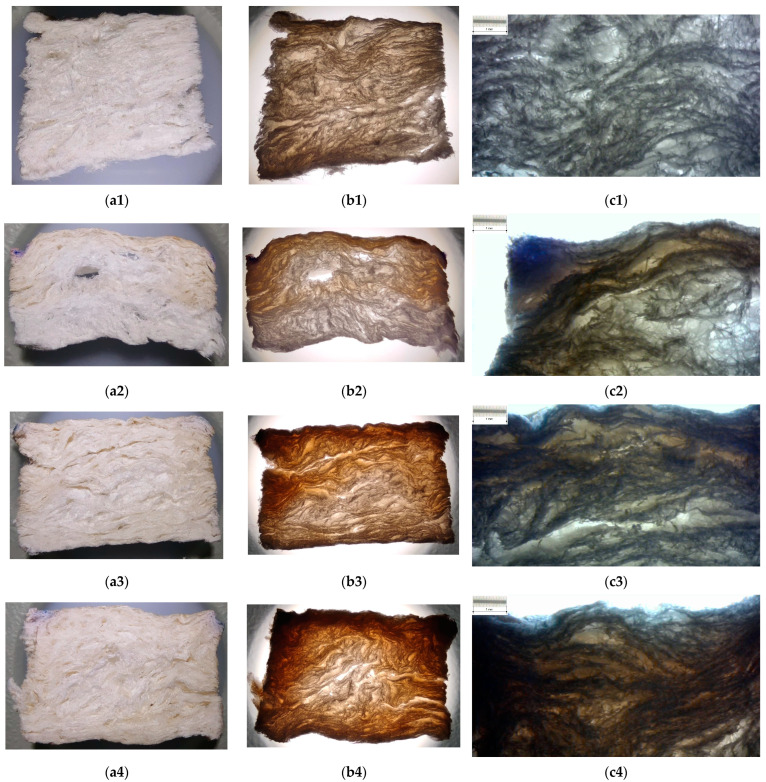
Images of longitudinal sections into samples based on 60 °SR beating degree: (**a**) photo on reflected light; (**b**) photo on transmitted light; (**c**) optical microphotograph of xylan–material interface (transmitted light, etalon scale marked on picture); (**1**) basic samples without xylan surface treatment; (**2**) samples with native xylan surface treatment; (**3**) samples with AKD hydrophobized xylan surface treatment; (**4**) samples with acetylated xylan surface treatment.

The results are depicted in Figure 6 for 43 °SR beating degree basic material and Figure 7 for 60 °SR beating degree basic material. For each microphotograph, an etalon scale (enabled by a standard calibration grid lamella) was suitably marked on the picture.

### 3.2. Static Water Contact Angle

**Figure 8:**
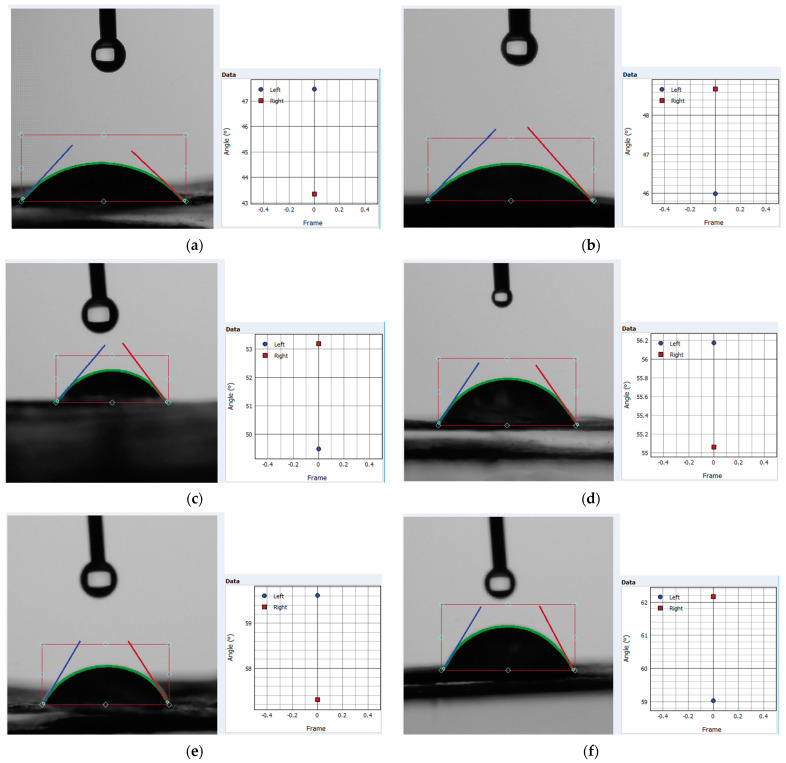
Snapshots within contact angle procedure for samples: (**a**) FL43PS10 with native xylan; (**b**) FL60PS10 with native xylan; (**c**) FL43PS10 with hydrophobized xylan; (**d**) FL60PS10 with hydrophobized xylan; (**e**) FL43PS10 with acetylated xylan; (**f**) FL60PS10 with acetylated xylan.

A set of snapshots within the contact angle evaluation procedure is presented in Figure 8, where the droplet on the sample surface can be observed, with marked left/right estimated angles through the image processing with data interpolation correlated procedure) and the angle distribution chart. The significance of each image is mentioned in the figure caption. According to the samples within Figure 8, Table 1 systematically presents values of each angle, average angle, and RMS errors of the estimation procedure (sample codes in Table 1 have the same meaning as those in Figure 5 (see Section 3.1)).

### 3.3. FT-IR Analysis

**Figure 9:**
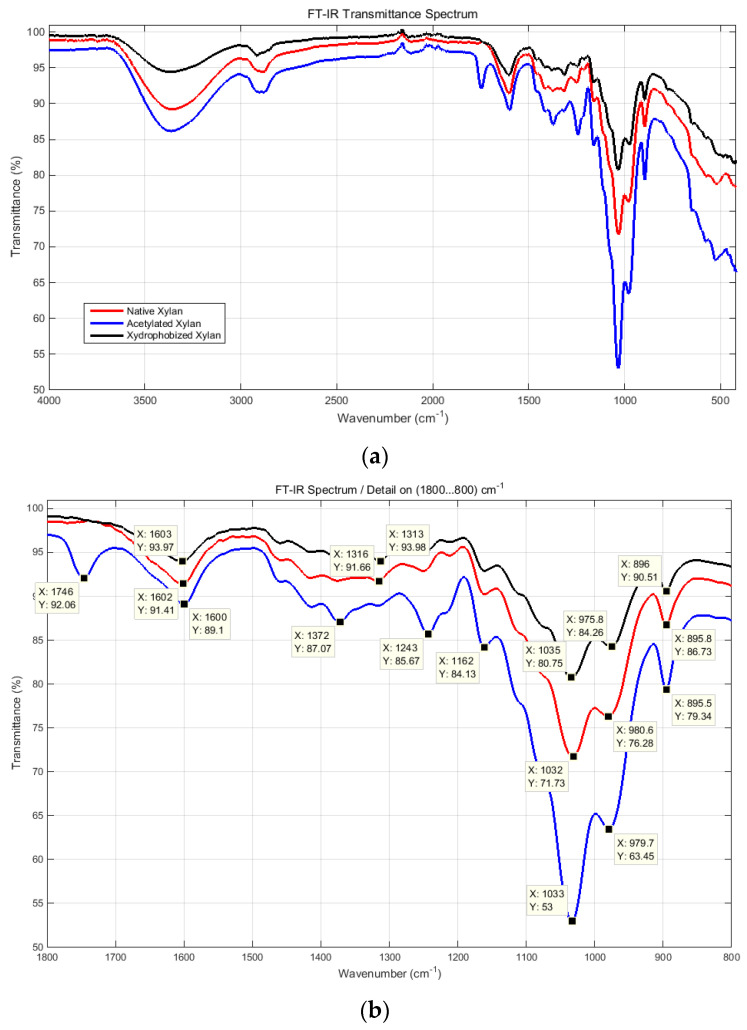

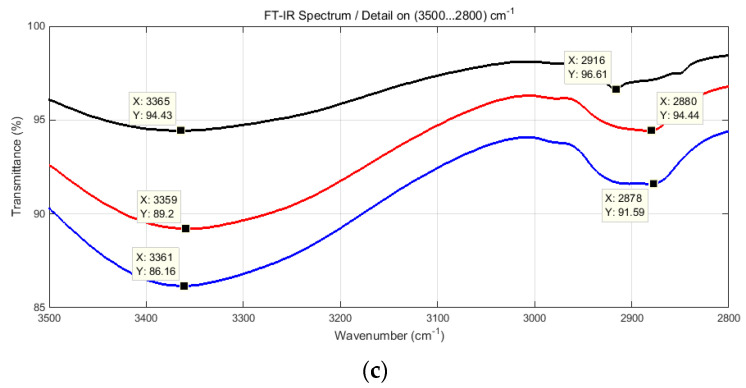
FT-IR spectra of softwood native xylan, acetylated xylan, and hydrophobized xylan: transmittance spectra of whole samples (**a**); detailed views with marked peaks (**b**,**c**).

The FT-IR spectral transmittance diagrams of xylan and xylan derivatives are presented in Figure 9. Compared with native xylan, the structural spectra of xylan derivatives indicated the presence of absorption peaks at 1746 cm^−1^, which are associated with C=O vibration stretching from acetyl and −COOH groups, and the vibration stretching characteristic absorption peaks of β-ketone ester bond formed between xylan hemicelluloses and AKD within 1602 cm^−1^ and 1733 cm^−1^ (please see details in Figure 9b,c).

### 3.4. Noise Insulation Characteristics

The investigations related to the noise insulation capability of xylan-based surface-treated foams were performed following the acoustic absorption coefficient, the acoustic reflection coefficient, and the surface acoustic impedance, with all of these supposing the normal sound incidence (according to the international standards and the experimental setup (see Section 2.5)). The raw data gained by experimental investigations were post-processed and managed using a Matlab^®^-based application in order to enable and facilitate comparative analyses between different categories of data.

**Figure 10:**
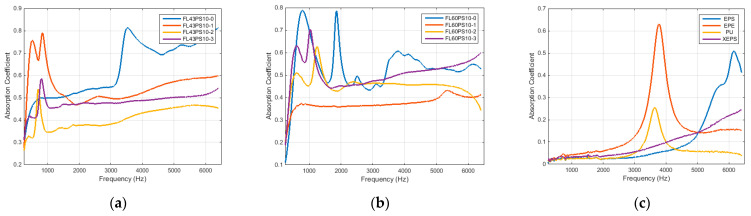
Acoustical absorption coefficient: (**a**) FL43PS10 basic material; (**b**) FL60PS10 basic material; (**c**) reference materials.

**Figure 11:**
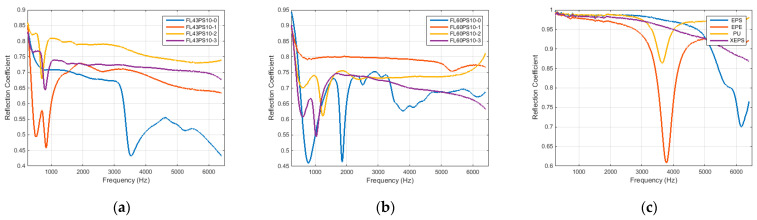
Acoustical reflection coefficient: (**a**) FL43PS10 basic material; (**b**) FL60PS10 basic material; (**c**) reference materials.

Graphs in Figure 10 present the results related to the acoustic absorption coefficient with respect to the frequency range enabled by the impedance tube. Diagrams were grouped by the basic material, such as 43 and 60 °SR beating degree, and reference petroleum-based materials. In the same manner, the results related to the acoustic reflection coefficient are presented in Figure 11.

**Figure 12:**
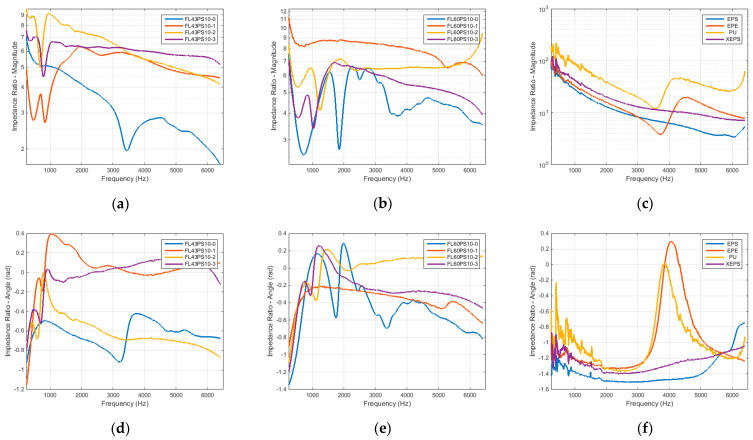
Surface acoustic impedance at normal sound incidence in terms of magnitude and angle: (**a**) FL43PS10 basic material—impedance magnitude; (**b**) FL60PS10 basic material—impedance magnitude; (**c**) reference materials—impedance magnitude; (**d**) FL43PS10 basic material—impedance angle; (**e**) FL60PS10 basic material—impedance angle; (**f**) reference materials—impedance angle.

Taking into account the previous grouping rule (FL43PS10, FL60PS10, and reference materials), the surface acoustic impedance is presented in Figure 12 in terms of the magnitude (using a vertical semi-logarithmic scale representation) and angle.

A comparative analysis of the effect induced by xylan-based treatments on the soundproofing ability of cellulose foams can be performed using the peak distribution of the acoustic absorption coefficient. Hereby, the maximum value and its corresponding frequency for each graph within Figure 10a,b is provided in Table 2.

## 4. Discussion

By analyzing dried samples photos in Figure 5, it can be observed that xylan treatments penetrate the foam based on cellulose fibers much more, with a low beating degree (between 40% for native xylan and 60% for acetylated xylan, of sample high), comparative with the foams from high beating degree cellulosic fiber (between 60% and 70%).

These aspects are also present on the sample kernel and become visible on the longitudinal section slices (please see photos in Figure 6 and Figure 7). Micrographs in Figure 6 and Figure 7 show the way that xylan penetrates the porous structure of the foam and fills the pore between cellulose fibers, as well as the depth of this penetration. It was observed that an increase in the depth at the same time as native xylan, hydrophobized xylan, and acetylated xylan were applied. Thus, the xylan derivatives intensively fill in the pores, and this fact can affect the noise insulation ability of treated foam, especially for high frequencies, but it is able to increase this ability for low–medium frequencies. On the other hand, they increase the surface structure integrity and decrease the wettability of the surface. The slice photos qualitatively indicate that acetylated xylan treatment is able to provide better functional properties than the hydrophobized xylan, both exceeding the capability of native xylan-based treatment.

An additional indicator of changing wettability can be provided by the static water contact angle. Values within Table 1 enable a quantitative view of the ability of xylan-based surface treatments to decrease foam wettability. The hierarchy (native, hydrophobized, acetylated) of xylan, in order to improve the hydrophobicity of samples, can also be observed in snapshots in Figure 8. But, the average angle values (see Table 1) clearly supply this order and reveal that foams based on 60 °SR beating degree fibers overrun those with lower beating degree. This aspect can be justified by the relative length of cellulose fiber and different fine percentages between the two raw fiber components.

Taking into account the main goal of this research, it has to evaluate the new soundproofing abilities of surface-treated samples in order to identify and characterize changes produced by the xylan fill-in effect. The main aspect is provided by the acoustic absorption coefficient (see diagrams in Figure 10). Let us talk distinctively about the two groups of foams.

Thus, the group based on FL43PS10 basic material presents the following characteristics: (a) all treatments shift the frequency peak to the lower values (under 1000 Hz) compared with the basic foam sample; (b) all treatments decrease the absorption ability for medium–high frequencies (beyond 2000 Hz), but provide an approximately constant value (α ≅ 0.4–0.6, depending to the treatment type); (c) native xylan presents smallest changings; (d) hydrophobized xylan presents the worst solution because of its major decreasing of absorption ability; (e) acetylated xylan presents a relative constant characteristic (α ≅ 0.45–0.50) for the whole frequency range, and, by demonstrating the better performances related to the wettability, this treatment represents the better solution for this foam type.

On the other hand, the group based on FL60PS10 basic material presents different characteristics as follows: (a) all treatments reject the frequency peak around 2000 Hz; (b) native xylan substantially decreases the absorption ability for the entire frequency range and, thus, represents the worst solution; (c) hydrophobized xylan slowly decrease the absorption, especially for high frequencies (greater than 3000 Hz); (d) acetylated xylan practically maintain the same characteristic trend than the surface untreated foam sample, thereby representing the better solution for this type of foam. By combining the acoustic absorption specificities of the two groups and supposing the previously presented wettability characteristics of each treatment type, it is shown that (a) native xylan can be used for low-level beating degree cellulose fibers but does not enable enough hydrophobicity contribution; (b) hydrophobized xylan represents a good choice for high-level beating degree cellulose fibers, especially for medium frequencies between 1000 and 5000 Hz; (c) acetylated xylan provide better treatment alternative at the same time with the increase in beating degree.

**Figure 13:**
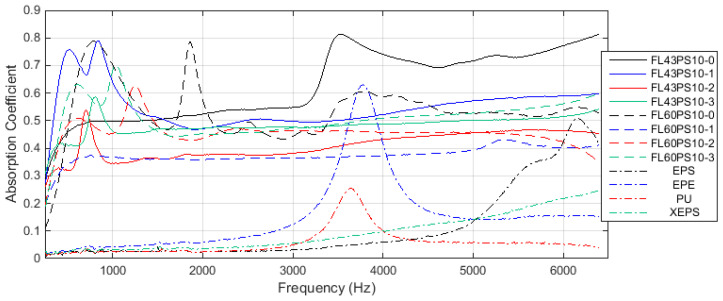
Comparative analysis of acoustic absorption coefficient (see text for coding details).

Compared with petroleum-based materials, good acoustic absorption performances of cellulose fiber-based foams (with or without surface treatments) are evident (please see diagrams within Figure 10). This fact and the previously presented specificities clearly result from the overlapped charts in Figure 13.

**Figure 14:**
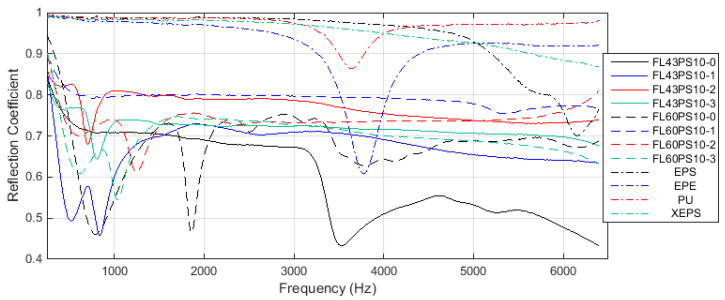
Comparative analysis of acoustic reflection coefficient (see text for coding details).

The relative decrease in absorption ability for treated samples, with respect to the basic foam structural characteristic and xylan derivatives, can be explained by a decrease in near-surface void number (through the xylan fill-in effect) and an increase in acoustic reflection coefficient. Thus, diagrams within Figure 11 clearly show this aspect. Additionally, the overlapped charts in Figure 14 reveal that commercially petroleum-based materials present maximum reflection abilities compared with cellulosic foams, but surface treatments increase, more or less, this foam’s ability (to the absorption detriment). A good choice of acetylated xylan-based treatment also results from the comparative diagram of the acoustic reflection coefficient (see Figure 14).

The diagrams of surface acoustic impedance at normal sound incidence (Figure 12) highlight previously presented aspects, especially in terms of impedance magnitude (impedance angle diagrams reveal the changes within magnitude evolutions). Greater values of petroleum-based materials’ impedance sustain the concluding remark that cellulose foams (with or without surface hydrophobicity treatments) enable good acoustic absorption properties compared with these large-scale used materials in soundproofing applications.

## 5. Conclusions

The authors developed three types of surface-treated cellulosic foam-formed materials based on native xylan and xylan derivatives in order to acquire a potential consistent enhancement of foam hydrophobicity property. This improvement enables the materials to be incorporated into practical applications within the area of noise insulation.

The results reveal that acetylated xylan provides optimal performances in terms of both maintaining the noise insulation capability at a suitable level and decreasing the wettability of the basic foam to a serviceable acceptable level, according to the practice requirements. Hydrophobized xylan presents a selective application range with respect to the raw cellulosic fibers’ characteristics and the incident noise frequency domain. On the other hand, native xylan does not strictly present a practical relevance due to its lowest capacity regarding the wettability protection of the external foam surface.

Future investigations will be performed in order to identify and evaluate other practical, suitable, and environmentally friendly solutions for surface treatments of cellulosic foam-formed materials with the highest performances in both soundproofing and additional required functional properties.

## Figures and Tables

**Table 1:** Values of contact angle for all samples xylan-based treated.

Sample *	Left Angle (°)	Right Angle (°)	Average Angle (°)	Left RMSE **	Right RMSE **
FL43PS10/xylan 1	47.46	43.34	45.4	0.89234076	0.968734416
FL60PS10/xylan 1	45.98	48.67	47.32	0.45422208	0.476735329
FL43PS10/xylan 2	49.47	53.17	51.32	0.81973641	0.772447342
FL60PS10/xylan 2	56.17	55.06	55.61	0.58340201	0.430664096
FL43PS10/xylan 3	59.61	57.3	58.45	0.52365381	0.441699407
FL60PS10/xylan 3	59.02	62.16	60.59	0.69721882	0.628683974

* See text for samples coding details; ** RMSE: Root Mean Square Error.

**Table 2:** Peak values of acoustic absorption coefficient (α) with respect to the frequency.

CF Material *	Parameter	Surface Treatment			
		No Xylan	Xylan 1 *	Xylan 2 *	Xylan 3 *
FL43PS10	Freq. (Hz)	3528	840	704	808
	α	0.8131	0.7902	0.5384	0.5856
FL60PS10	Freq. (Hz)	792	5368	1248	1040
	α	0.7887	0.4325	0.6277	0.7029

* CF: Cellulose-based foam; Xylan 1: native xylan; Xylan 2: hydrophobized xylan; Xylan 3: acetylated xylan.

## Data Availability

Data are contained within the article.
